# Dosimetric impact of deformable image registration using radiophotoluminescent glass dosimeters with a physical geometric phantom

**DOI:** 10.1002/acm2.13890

**Published:** 2023-01-07

**Authors:** Siwaporn Sakulsingharoj, Noriyuki Kadoya, Shohei Tanaka, Kiyokazu Sato, Mitsuhiro Nakamura, Keiichi Jingu

**Affiliations:** ^1^ Department of Radiation Oncology Tohoku University Graduate School of Medicine Sendai Japan; ^2^ Division of Radiation Oncology, Faculty of Medicine Ramathibodi Hospital Mahidol University Bangkok Thailand; ^3^ Department of Radiation Technology Tohoku University Hospital Sendai Japan; ^4^ Department of Radiation Oncology and Image‐Applied Therapy Kyoto University Kyoto Japan; ^5^ Department of Information Technology and Medical Engineering, Human Health Sciences, Graduate School of Medicine Kyoto University Kyoto Japan

**Keywords:** adaptive radiotherapy, deformable image registration, dose accumulation, phantom, radiotherapy

## Abstract

**Purpose:**

To study the dosimetry impact of deformable image registration (DIR) using radiophotoluminescent glass dosimeter (RPLD) and custom developed phantom with various inserts.

**Methods:**

The phantom was developed to facilitate simultaneous evaluation of geometric and dosimetric accuracy of DIR. Four computed tomography (CT) images of the phantom were acquired with four different configurations. Four volumetric modulated arc therapy (VMAT) plans were computed for different phantom. Two different patterns were applied to combination of four phantom configurations. RPLD dose measurement was combined between corresponding two phantom configurations. DIR‐based dose accumulation was calculated between corresponding two CT images with two commercial DIR software and various DIR parameter settings, and an open source software. Accumulated dose calculated using DIR was then compared with measured dose using RPLD.

**Results:**

The mean ± standard deviation (SD) of dose difference was 2.71 ± 0.23% (range, 2.22%–3.01%) for tumor‐proxy and 3.74 ± 0.79% (range, 1.56%–4.83%) for rectum‐proxy. The mean ± SD of target registration error (TRE) was 1.66 ± 1.36 mm (range, 0.03–4.43 mm) for tumor‐proxy and 6.87 ± 5.49 mm (range, 0.54–17.47 mm) for rectum‐proxy. These results suggested that DIR accuracy had wide range among DIR parameter setting.

**Conclusions:**

The dose difference observed in our study was 3% for tumor‐proxy and within 5% for rectum‐proxy. The custom developed physical phantom with inserts showed potential for accurate evaluation of DIR‐based dose accumulation. The prospect of simultaneous evaluation of geometric and dosimetric DIR accuracy in a single phantom may be useful for validation of DIR for clinical use.

## 1 INTRODUCTION

Deformable image registration (DIR) plays an important role in modern radiotherapy including image‐guided radiotherapy (IGRT) and adaptive radiotherapy (ART). DIR can be used at planning and during IGRT for automatic segmentation^1^, ^2^, ^3^, ^4^, ^5^, ^6^ and multimodality image fusion to reduce the delineation workload. Deep learning‐based segmentation techniques have been used in radiotherapy, since it represents a significant potential to expedite the contouring process and improve contour consistency.^7^ DIR is also used to determine the accumulated dose during the course of radiotherapy for dose reporting and ART.^8^, ^9^, ^10^, ^11^ Several studies have reported that DIR accuracy depends on the DIR software, algorithm, parameter settings, and procedures.^12^, ^13^, ^14^, ^15^ At present, there are many commercially available DIR software programs in clinical practice, including Velocity AI (Varian Medical Systems, Palo Alto), RayStation (RaySearch Laboratories, Stockholm, Sweden) and MIM Software (MIM Software Inc, Cleveland).^13^, ^16^, ^17^ Velocity used intensity‐based DIR, and structure‐based DIR. RayStation used hybrid intensity‐based and structure‐based DIR, and biomechanically based DIR. MIM Software used intensity‐based DIR, and hybrid intensity‐based and structure‐based DIR. Therefore, validation of DIR accuracy is necessary in radiotherapy prior to its clinical use because uncertainties and errors in the registration transformation may cause incorrect dose distribution determinations. Previous studies^18^, ^19^ have reported that intensity‐based DIR had inferior performance than hybrid DIR in both Dice similarity coefficient (DSC) and accumulated dose error. These results suggested that DIR accuracy affected the accuracy of the accumulated dose.

The American Association of Physicists in Medicine (AAPM) Task Group No. 132 (TG 132) has recommended end‐to‐end physical phantom tests in order to ensure accurate data representation, image transfer, and integrity verification between image acquisition devices, image registration systems, and other radiotherapy systems that use the image registration results for treatment computation or plan modification.^20^ Several studies have reported the development of custom physical phantoms to evaluate geometric DIR accuracy.^16^, ^21^, ^22^ These studies have evaluated only geometric DIR accuracy, but such evaluation is insufficient for clinical practice because it must also consider dosimetric DIR accuracy.^23^ Especially in the case of DIR applications for dose mapping and accumulation, the need for accurate validation is not limited to the evaluation of geometric accuracy. In fact, the need to account for DIR‐related dosimetric inaccuracies represents a key area in the field of ART.^24^ Therefore, both image and dose deformations should be evaluated in determinations of DIR accuracy. Regarding dosimetric evaluation, a composite imaging/dosimetry end‐to‐end phantom as a good example to check the dose delivery accuracy of an intrafraction image guidance system.^25^ A deformable gel phantom^26^, ^27^ could be used to measure the cumulative dose. In those studies, the gamma index was used to evaluate dose accumulation accuracy given the measured dose distribution. However, such a phantom requires special knowledge and materials. Moreover, gel phantom did not have anatomical site and fiducial marker. Thus, this phantom could not be used to evaluate geometric DIR accuracy using DSC and target registration error (TRE).

To date, no study has evaluated both geometric and dosimetric DIR accuracy in the same phantom. This study aims to evaluate both geometric and dosimetric DIR accuracy using custom developed phantom with various inserts in which a small dosimeter can be inserted for dose measurement. This phantom can be used to evaluate both geometric and dosimetric DIR accuracy.^17^ Radiophotoluminescent glass dosimeters (RPLDs) were selected to measure dose because of their many advantages such as small detector size, good reproducibility, small effective readout area, wide measurable dose range, repeatable readout, and small fading effect.^28^ Although several previous studies have evaluated dose accumulation based on DIR,^18^, ^29^, ^30^ none of these studies has used RPLDs for evaluation of measured dose accumulation. The present study aims to evaluate the dosimetric impact of DIR using RPLD and custom developed phantom with various inserts.

## 2 MATERIALS AND METHODS

### 2.1 RPLD dosimetry

AGC model GD‐302 M RPLDs (AGC Techno Glass Corp., Shizuoka, Japan) were used in this study. The shape of this model is cylindrical with a diameter of 1.5 mm and a length of 12 mm. The glass dosimeter's density was 2.61 g/cm^3^, and its effective atomic number was 12.039.^31^ The RPLD calibration factor was calculated as the absorbed dose (D_ref_) divided by the RPLD reading. The D_ref_ was measured with a 0.6 cm^3^ PTW farmer chamber (30013, PTW, Freiburg, Germany) inserted into tough water phantom at a depth of 10 cm and irradiated with a field size 10 × 10 cm^2^ using 10 MV X‐rays. The RPLD and PTW 30013 farmer measurements were performed using same setup geometry. RPLD measurement was repeated two times. The RPLD could be repeatedly read in FGD‐1000 reader (AGC Techno Glass Corp., Shizuoka, Japan). A dataset had five consecutive readings. Three repeat readings were performed for the reproducibility of the RPLDs readings. After each reading the position of the RPLDs in the readout magazine was checked, as during the reading process the readout magazine was moved and there was a possibility for the RPLDs to rotate. The standard deviation (SD) of the mean of three readings was calculated.

### 2.2 Phantom design

**FIGURE 1:**
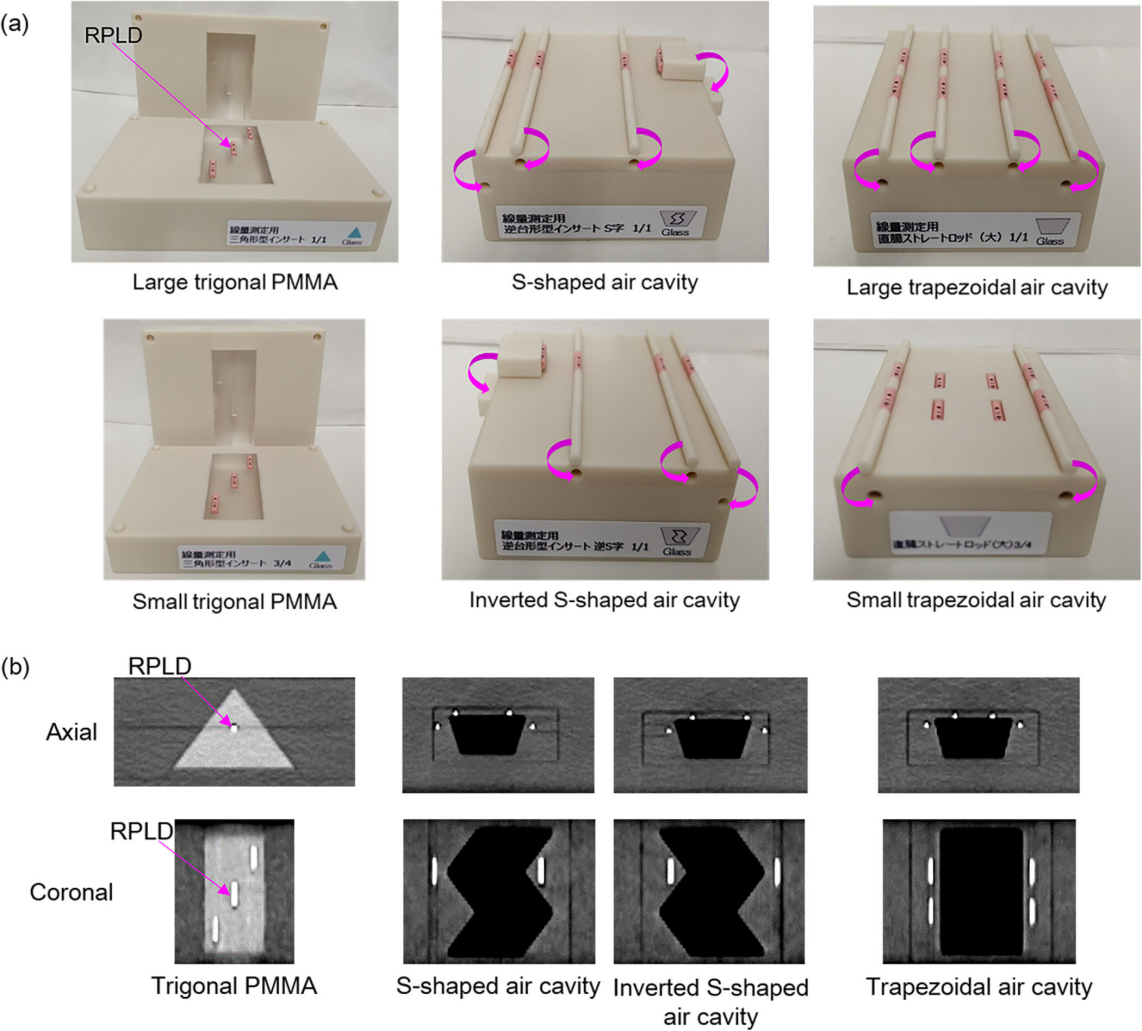
Overview of new custom insert with glass dosimeter. (a) Tumor‐proxy and rectum‐proxy custom inserts. (b) computed tomography (CT) images of tumor‐proxy and rectum‐proxy custom inserts

The physical phantom configuration with various inserts proposed in this study is an improved version of the phantom originally developed and reported by Kadoya et al.^17^ The physical size of improved phantom was same as the original phantom. The physical size of large phantom had a length, width, and height of 30, 8, and 15 cm, respectively. The physical size of small phantom had a length, width, and height of 22.5, 8, and 11.5 cm, respectively. We further developed new custom inserts into which RPLDs were inserted to measure dose accumulation (Figure 1). The new tumor‐proxy custom insert was designed to be split in half so that RPLDs could be tightly inserted within it. These custom trigonal polymethyl methacrylate (PMMA) inserts were of two different sizes: large for the large base phantom and small for the small base phantom. The new rectum‐proxy custom insert was made with small holes for inserting RPLDs. Four custom inserts were constructed: an S‐shaped air cavity, an inverted S‐shaped air cavity, a large trapezoidal air cavity for the large base phantom, and a small trapezoidal air cavity for the small base phantom. Three RPLDs can be placed in each trigonal PMMA custom insert. Four RPLDs can be inserted in the S‐shaped air cavity custom insert and the inverted S‐shaped air cavity custom insert. Eight RPLDs can be inserted in each trapezoidal air cavity custom insert.

Three phantom configurations were applied to the large phantom. Phantom configurations 1, 2, and 3 consisted of octagonal tough bone custom inserts in slots 1 and 3, a large trigonal PMMA custom insert in slot 2, and tough water custom inserts in slots 4 and 6. The air cavity custom insert in slot 5 was a different shape for each phantom configuration: an S‐shaped air cavity custom insert in phantom configuration 1, a trapezoidal air cavity custom insert in phantom configuration 2, and an inverted S‐shaped air cavity custom insert in phantom configuration 3. Phantom configuration 4, the only phantom configuration for the small phantom, used the same six custom inserts as in phantom configuration 2.

**FIGURE 2:**
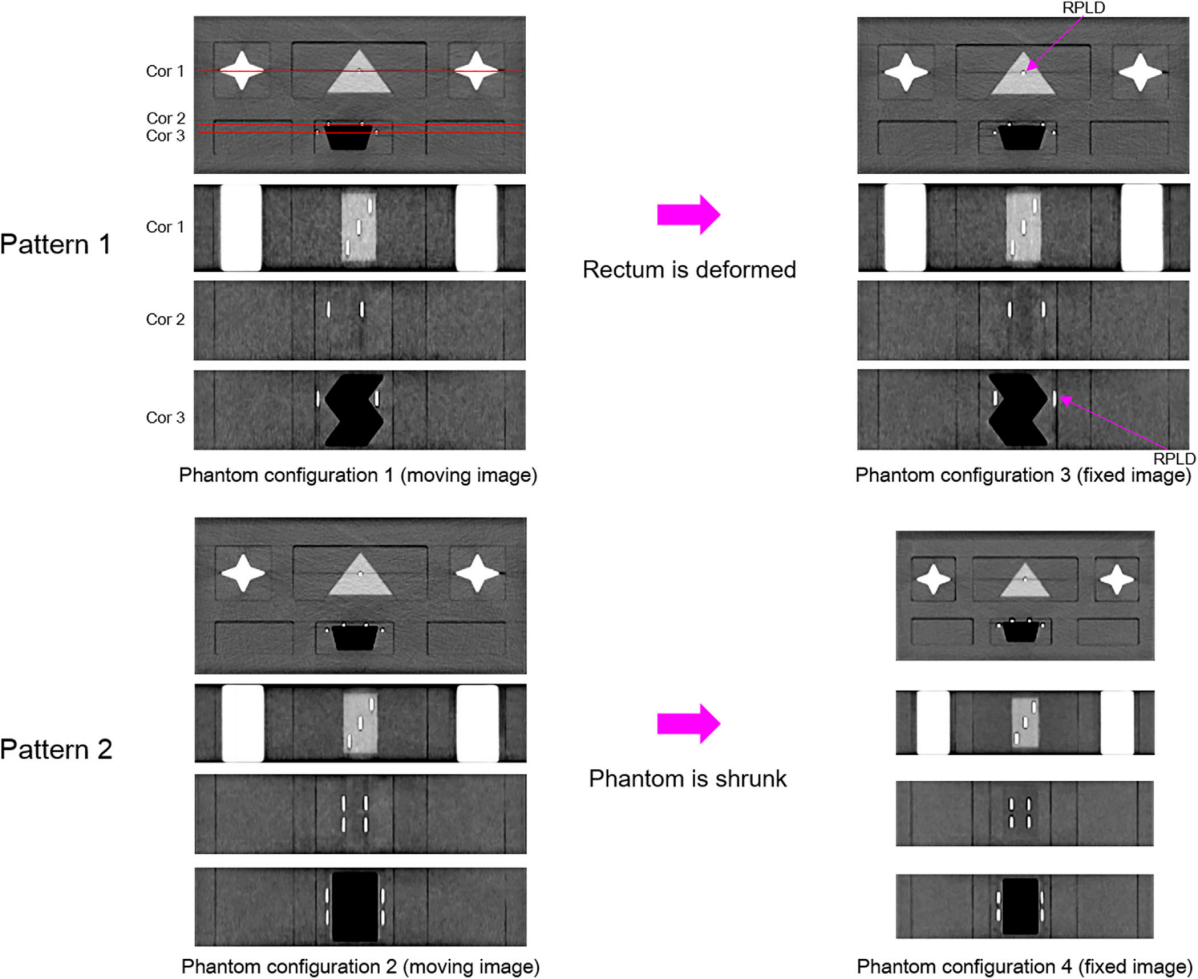
Overview of configuration combination for each of the two deformable image registration (DIR) patterns

The computed tomography (CT) images of the phantom were acquired with four different configurations by a SOMATOM Definition AS+ (Siemens Healthineers, Forchheim, Germany). The images were acquired using helical scans, a tube voltage of 120 kVp, a slice thickness of 2 mm, and a voxel size of 0.98 mm × 0.98 mm × 2 mm. Two DIR patterns were applied to combinations of the four phantom configurations (Figure 2). For DIR pattern 1, phantom configuration 3 (fixed image) was combined with phantom configuration 1 (moving image), the rectum‐proxy was deformed owing to the different rectum‐proxy shapes in the two configurations, and the positions and number of RPLDs were kept consistent between phantom configurations 3 and 1 for combined dose. For DIR pattern 2, the phantom was shrunk; phantom configuration 4 (fixed image) was combined with phantom configuration 2 (moving image), the whole phantom was deformed owing to the different phantom sizes in the two configurations, and the positions and number of RPLDs were kept consistent between phantom configurations 4 and 2 for combined dose.

Phantom configurations 1 and 2 (moving image) are phantom configurations 1 and 2 emulated a moving image. Phantom configurations 3 and 4 (fixed image) are phantom configurations 3 and 4 emulated a fixed image.

### 2.3 Treatment planning system

In our treatment planning system process, we designed four volumetric modulated arc therapy (VMAT) plans for each of the four phantom configurations. We made two different plans to simulated re‐plan case in rectum‐proxy shape change. We created new plan for tumor‐proxy and rectum‐proxy shrinkage and made two different plans. We used four different VMAT plans to investigate impact of tumor‐proxy and rectum‐proxy changed size and shape. In clinical practice, DIR‐based dose accumulation was calculated using initial plan and re‐plan for the tumor and organ at risk change in inter‐fractions. In this study, we evaluated with different plans based on clinical practice. In each case, planning was generated as one full arc (VMAT technique) with 10 MV X‐rays. The 200 cGy dose is prescribed to tumor‐proxy (2 cm planning target volume (PTV) margin) with 100% coverage of 95% of the target volume. The large distance between the tumor‐ and rectum‐proxy in the physical phantom caused the dose to be lower in the rectum‐proxy than in clinical practice. Thus, it was necessary to plan a large PTV margin. The treatment planning was performed using Varian Eclipse treatment planning version 13.5 with Acuros XB Algorithm (Varian Medical Systems, Palo Alto).

### 2.4 Dose measurement

**FIGURE 3:**
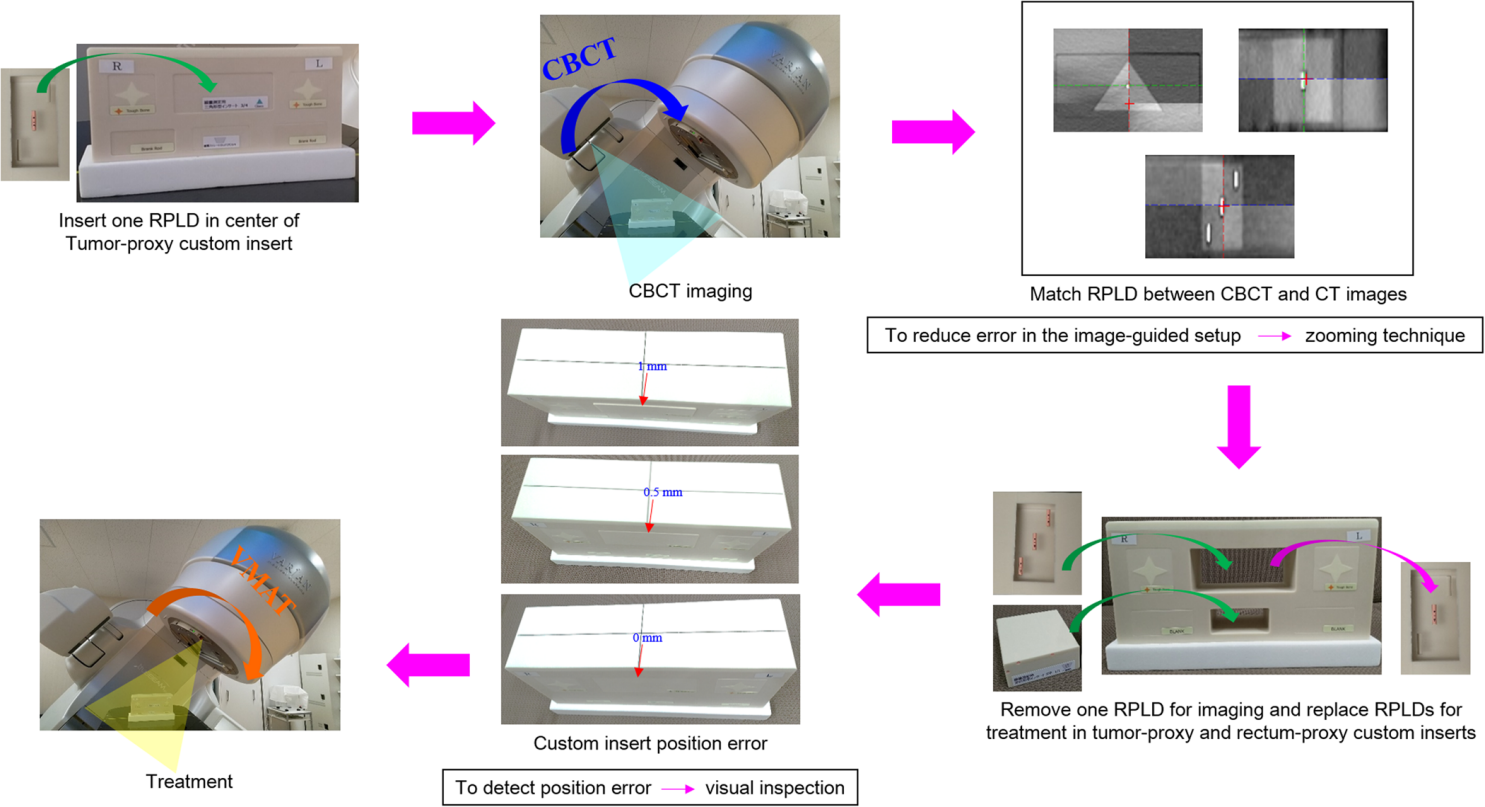
Process workflow for dose measurement for each phantom configuration

**FIGURE 4:**
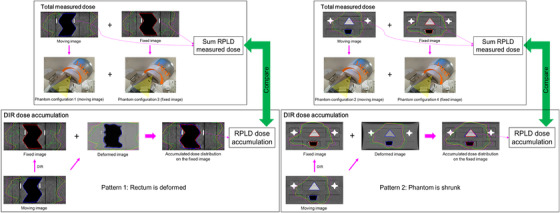
Process of comparison of total measured dose and deformable image registration (DIR)‐based dose accumulation. The total measured dose is the sum of the radiophotoluminescent glass dosimeter (RPLD) dose for one phantom configuration (fixed image) and the RPLD dose for the other phantom configuration (moving image). The DIR‐based dose accumulation is calculated as the spatial average of the sum of the dose over the area enclosed by the RPLD contour for fixed image and the dose from moving image

A process workflow for dose measurement of each phantom configuration is shown in Figure 3. First, one RPLD was placed in the center of the tumor‐proxy custom insert for a cone beam computed tomography (CBCT) confirmation of RPLD position for imaging. Next, the RPLD was matched between the CT and CBCT images. A zooming technique was used to reduce error in the image‐guided setup because this technique magnified an image that the details in the image became more visibly and clearly. The one RPLD for imaging was removed and RPLDs for treatment were then replaced in the tumor‐proxy and rectum‐proxy custom inserts for each phantom configuration and the VMAT plan was delivered using a TrueBeam (Varian Medical Systems, Palo Alto, USA). We used two RPLD sets: (1) the first RPLD set for imaging and (2) the second RPLD set for treatment. Custom insert position error was possible but was easy to detect by visual inspection. Therefore, we must carefully pay attention to the custom insert position. RPLD measurement was repeated three times for each VMAT plan and each RPLD dose was averaged. The total measured dose was the sum of the RPLD dose for one phantom configuration (fixed image) and the RPLD dose for the other phantom configuration (moving image) (Figure 4). For example, DIR pattern 1 in the tumor‐proxy custom insert entailed three such sums, one for each of the three RPLDs: RPLD dose at superior position for phantom configuration 3 + RPLD dose at superior position for phantom configuration 1 = total RPLD dose at superior position; RPLD dose at center position for phantom configuration 3 + RPLD dose at center position for phantom configuration 1 = total RPLD dose at center position; and RPLD dose at inferior position for phantom configuration 3 + RPLD dose at inferior position for phantom configuration 1 = total RPLD dose at inferior position.

### 2.5 Various DIR parameter settings

We used two types of commercially available DIR software in this study. The software packages were Velocity AI version 3.2.0 (Varian Medical Systems, Palo Alto, USA) and RayStation version 6.2 (RaySearch Laboratories, Stockholm, Sweden). Table 1 summarizes the DIR software, algorithms, and parameter settings, each of which was selected for this study because it is used in clinical practice. In Velocity, we used six DIR parameter settings with three DIR algorithms: “deformable multi pass (intensity‐based DIR),” “extended deformable multi pass (intensity‐based DIR),” and “structure guided deformable (structure‐based DIR)” with tumor and rectum. Two common situations occur in clinical practice: DIR is performed over the entire body or else only within some specific organs, for example, the tumor and rectum. Therefore, we used two field of views (FOVs) in this study: (1) cover the whole phantom and (2) cover the only tumor and rectum. The deformable multi pass algorithm uses a 3‐pass (low–medium–high resolution) deformable registration that yields finer touch‐up. The extended deformable multi pass algorithm uses a 6‐pass deformable registration, going into finer resolution than the deformable multi pass. The structure guided deformable algorithm uses a hybrid registration of the deformable multi pass and structure outlines to influence the deformable registration.^32^ In RayStation, we used four DIR parameter settings with one DIR algorithm (hybrid intensity and structure‐based DIR), one grid size (2.5 mm × 2.5 mm × 2.5 mm), two region of interests (ROIs) (controlling and focus ROIs), and two methods using structures: (1) whole body and (2) tumor and rectum. The hybrid intensity and structure based algorithm combined image intensity information with anatomical information as provided by contoured image sets.^33^

**TABLE 1:** Summary of DIR software packages, algorithms and parameter settings

DIR parameter setting	DIR software (version)	DIR algorithm	FOV	Grid size (mm3)	Structure
1	Velocity (3.2.0)	Deformable multi pass	Cover whole phantom	Unspecified	–
2		Extended deformable multi pass			–
3		Structure guided deformable			Tumor and rectum
4		Deformable multi pass	Cover only tumor and rectum	Unspecified	–
5		Extended deformable multi pass			–
6		Structure guided deformable			Tumor and rectum
7	RayStation (6.2)	Hybrid intensity and structure based	Unselected	2.5 × 2.5 × 2.5	Body (Controlling ROI)
8					Tumor and rectum (Controlling ROI)
9					Body (Focus ROI)
10					Tumor and rectum (Focus ROI)

Abbreviations: DIR, deformable image registration; FOV, field of view; ROI, region of interest.

Additionally, we used one type of an open source DIR software. The elastix is based on the well‐known open source Insight Segmentation and Registration Toolkit developed at the University Medical Center Utrecht.^34^ The software allows the user to easily set various parameters and is widely used in research on DIR techniques for medical images.^35^, ^36^ In this study, we used one parameter setting which was publicly available in the elastix website.^37^

### 2.6 DIR‐based dose accumulation

The planning CT images set for one phantom configuration (moving image) were deformed to the planning CT images set for the other phantom configuration (fixed image) by applying various DIR parameter settings (Table 1) to DIR of the two patterns (Figure 2). The moving image dose distribution was transformed to the fixed image, yielding a combined dose distribution on the fixed image. The DIR‐based dose accumulation was then calculated from the combined dose as spatially averaged across the fixed image RPLD contour (Figure 4).

### 2.7 Evaluation

The dosimetric accuracy of DIR was analyzed via quantitative evaluation of DIR‐based dose accumulation using absolute dose difference. The absolute dose difference between the measured dose and the DIR‐based dose accumulation was calculated by subtracting the total measured dose from the DIR‐based dose accumulation and dividing by the total measured dose.

The geometric accuracy of DIR was measured using DSC and mean distance to agreement (MDA) for the tumor‐proxy and rectum‐proxy contours. TRE was used at the centroid of the RPLD contour because of the dependence of DSC calculations on structure volume^20^ and the small size of RPLD contours. DSC can be poor even for a small shift in small structure volume.

DSC is designed to evaluate the quantitative volumetric overlap between the reference contour and the deformed contour. DSC was calculated as

(1) $$\mathrm{DSC}=2\left(V_{d}\cap V_{r}\right)/\left(V_{d}+V_{r}\right),$$

where V_r_ is the volume of the reference contour and V_d_ is the volume of the deformed contour. A DSC value of 0 indicates no spatial overlap, while a value of 1 indicates perfect coincidence between the two contoured volumes. The MDA is the mean surface distance, across all points, from each point in the reference contour to the closest point in the deformed contour. TRE is the spatial discrepancy between the centroid positions of the reference contour and the deformed contour. TRE was defined as

(2) $$\mathrm{TRE}=\left[{\left(X_{r}-X_{d}\right)}^{2}+{\left(Y_{r}-Y_{d}\right)}^{2}+{\left(Z_{r}-Z_{d}\right)}^{2}\right]{}^{1/2},$$

where X is the sagittal plane, Y is the coronal plane, Z is the transverse plane, r is the reference contour, and d is the deformed contour.

The relationship between geometric and dosimetric accuracy of DIR was analyzed by using Pearson correlation coefficient (*r*) to measure the correlation of absolute dose difference with DSC, MDA, and TRE.

## 3 RESULTS

**FIGURE 5:**
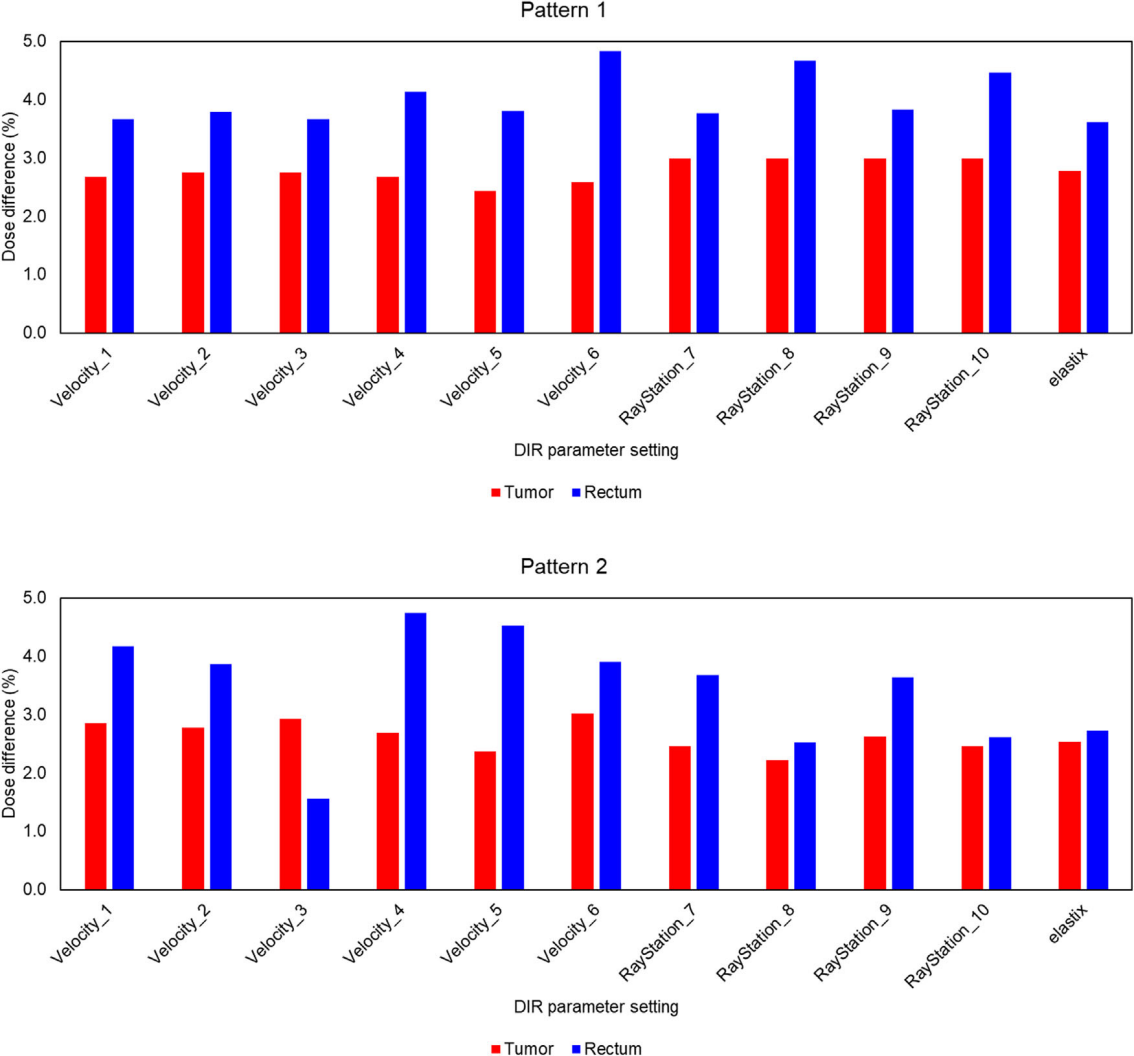
Summary of DIR‐based dose accumulation error for all data

The mean ± SD of absolute dose difference between the measured dose and the calculated dose (without DIR) was a 2.97 ± 0.81% (range, 2.74%–3.15%) for tumor‐proxy and 3.75 ± 0.78% (range, 2.88%–4.51%) for rectum‐proxy. Figure 5 shows a summary of the absolute dose difference between the measured dose and the DIR‐based dose accumulation (DIR‐based dose accumulation error) for all DIR parameter settings. The mean ± SD of the DIR‐based dose accumulation error was 2.71 ± 0.23% (range, 2.22%–3.01%) for tumor‐proxy and 3.74 ± 0.79% (range, 1.56–4.83%) for rectum‐proxy, indicating that the DIR‐based dose accumulation error was approximately 3% for tumor‐proxy and less than 5% for rectum‐proxy. The mean SD value of the reproducibility of the RPLDs readings was 0.18%.

**FIGURE 6:**
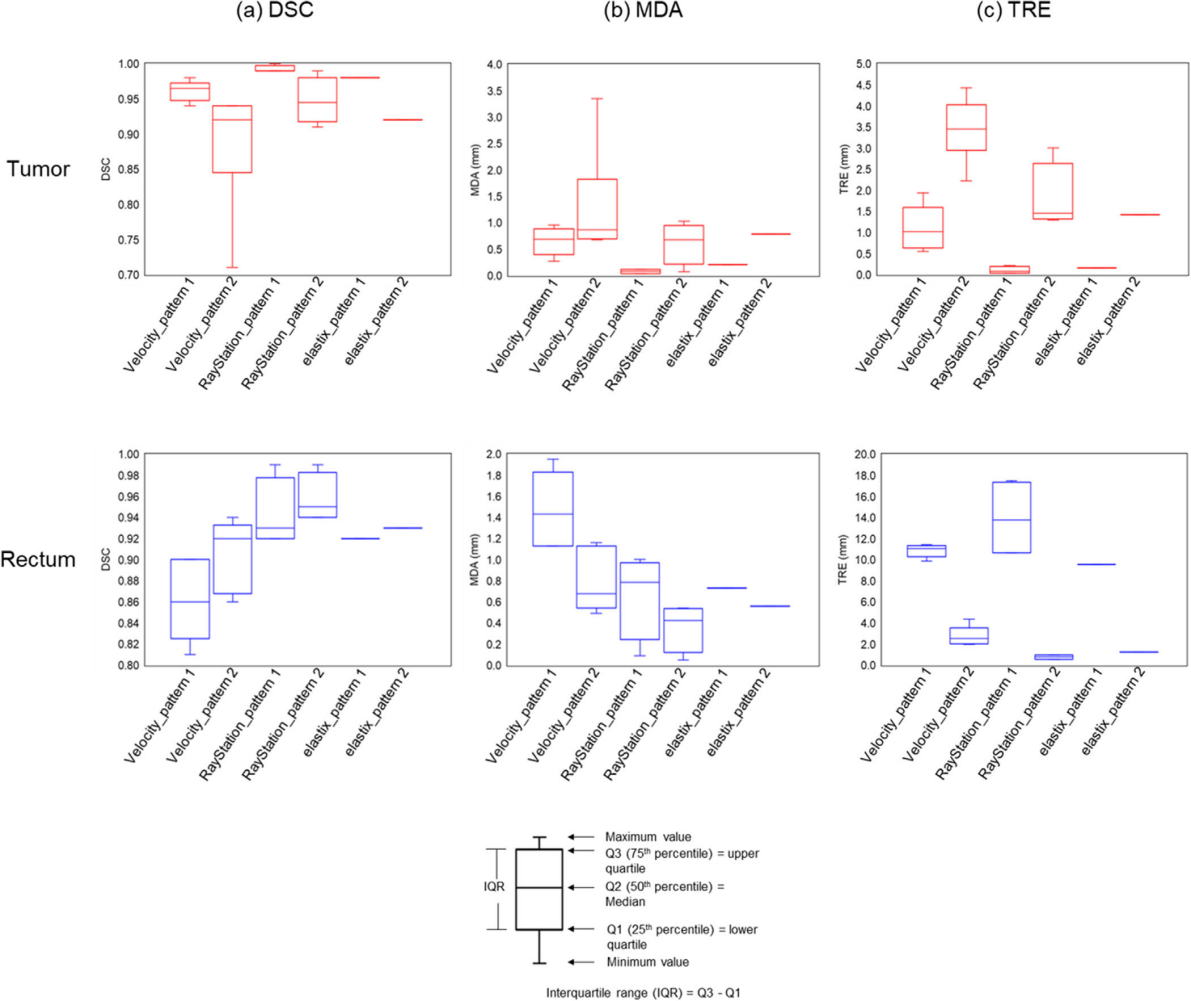
Box plots of (a) Dice similarity coefficient (DSC), (b) mean distance to agreement (MDA), and (c) target registration error (TRE) for all data

A summary of DSC, MDA, and TRE results, encompassing various DIR parameter settings, is shown in Figure 6. The mean ± SD of all metrics for tumor‐proxy was 0.94 ± 0.06 (range, 0.71–1.00) for DSC, 0.70 ± 0.70 mm (range, 0.03–3.35 mm) for MDA, and 1.66 ± 1.36 mm (range, 0.03–4.43 mm) for TRE. The mean ± SD of all metrics for rectum‐proxy was 0.91 ± 0.05 (range, 0.81–0.99) for DSC, 0.86 ± 0.50 mm (range, 0.05–1.95 mm) for MDA, and 6.87 ± 5.49 mm (range, 0.54–17.47 mm) for TRE. These results suggested that DIR accuracy had a wide range among DIR parameter setting.

In terms of the influence of the DIR accuracy on DIR‐based dose accumulation error. For tumor‐proxy, the best DIR accuracy value (DIR‐based dose accumulation error value) was 1.00 (2.99%) for DSC, 0.03 mm (2.99%) for MDA, and 0.03 mm (2.99%) for TRE and the worst DIR accuracy value (DIR‐based dose accumulation error value) was 0.71 (2.93%) for DSC, 3.35 mm (2.93%) for MDA, and 4.43 mm (2.77%) for TRE, showing that the DIR accuracy had a large variation between good and poor DIR parameter setting but the DIR‐based dose accumulation error was similar. For rectum‐proxy, the best DIR accuracy value (DIR‐based dose accumulation error value) was 0.99 (4.67%) for DSC, 0.05 mm (2.52%) for MDA, and 0.54 mm (3.64%) for TRE and the worst DIR accuracy value (DIR‐based dose accumulation error value) was 0.81 (4.13%) for DSC, 1.95 mm (4.13%) for MDA, and 17.47 mm (4.67%) for TRE, indicating that the DIR accuracy was wide range between good and poor DIR parameter setting but the DIR‐based dose accumulation error was slightly different.

**FIGURE 7:**
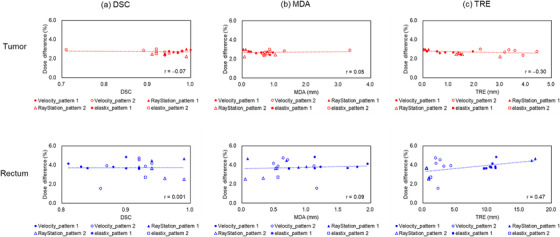
Correlations between all metrics with DIR‐based dose accumulation error for all data (a) Dice similarity coefficient (DSC), (b) mean distance to agreement (MDA), and (c) target registration error (TRE)

A summary of correlations between all metrics with the DIR‐based dose accumulation error for all DIR parameter settings is shown in Figure 7 (a) DSC, (b) MDA, and (c) TRE.

## 4 DISCUSSION

In this study evaluated DIR‐based dose accumulation results with RPLD measurements using new custom inserts in the physical geometric phantom. We used two combinations of phantom configuration and three different DIR software with various DIR parameter settings to study its geometric and dosimetric DIR accuracy.

RPLDs have the advantage of feasible use for in vivo dosimetry^31^ and postal dosimetry audits for intensity‐modulated radiation therapy (IMRT).^38^ Hsu et al.^31^ assessed the potential applications of RPLDs for in vivo high‐dose‐rate (HDR) brachytherapy dose verification. They concluded that for HDR brachytherapy in vivo dosimetry, RPLDs were reliable dosimeters with clinically acceptable with 5%. Okamoto et al.^38^ established an efficient postal audit system in IMRT using RPLDs in Japan. Their results showed that an audit system using RPLDs was a high‐accuracy system with a high‐level criterion of 3%. Thus, RPLDs were considered reliable and suitable for dose verification in the present study. The response of glass dosimeter irradiated with photon beams had energy dependence. Several studies have reported that the results of energy dependence study were presented as the relevant correction factors.^38^, ^39^, ^40^ Therefore, the calibration factor of RPLD and VMAT treatment in this study were used with the same energy photon beam (10 MV). If the calibration factor of RPLD and VMAT treatment were used with the different energy photon beam, the results may be change. To compare, the readout reproducibility of RPLDs reported by Wesolowska PE et al.^40^ had the SD value of 0.16%. This result is consistent with our result.

The absolute dose difference between the measured dose and the calculated dose (without DIR) showed a range of 2.74 to 3.15% for tumor‐proxy and 2.88 to 4.51% for rectum‐proxy. Okamoto et al.^38^ reported a range of dose difference between measurement (RPLD) and calculation of −2.9 to 2.8% for the PTV and −30.6% to 17.6% for the organ at risk. Yamazaki et al.^28^ showed that the dose differed by less than 5% between measurement (RPLD) and calculation for PTV. These results showed that the dose difference observed in our study between the measured dose and the calculated dose (without DIR) was within 5%, which was clinically acceptable and consistent with previous studies.^28^, ^38^ Based on this affirmation, the data we observed were considered reliable for dose evaluation in our study. Regarding dosimetric DIR accuracy, the DIR‐based dose accumulation error for all data showed a range of 2.22% to 3.01% for tumor‐proxy and 1.56% to 4.83% for rectum‐proxy. These data were similar results compare with simple condition without DIR. Thus, our DIR‐based dose accumulation error was reasonable. The DIR‐based dose accumulation error of rectum‐proxy was higher than that of tumor‐proxy because the rectum‐proxy had a lower dose than the tumor‐proxy.

Regarding the impact of the DIR accuracy on DIR‐based dose accumulation error. For tumor‐proxy, the DIR accuracy of TRE value (DIR‐based dose accumulation error value) was 0.03 mm (2.99%) for good DIR parameter setting and 4.43 mm (2.77%) for poor DIR parameter setting. For rectum‐proxy, the DIR accuracy of TRE value (DIR‐based dose accumulation error value) was 0.54 mm (3.64%) for good DIR parameter setting and 17.47 mm (4.67%) for poor DIR parameter setting. These results suggested that the DIR accuracy changed according to the DIR parameter setting. Several studies have evaluated DIR accuracy using many DIR algorithm.^13^, ^14^, ^17^, ^41^ These studies showed that the DIR accuracy strongly depended on the DIR algorithm and procedure. Our result was consistent with their studies. However, our results indicated that the DIR‐based dose accumulation error was a slight difference with different DIR parameter setting. Although the DIR accuracy depended on the DIR parameter setting, but the DIR‐based dose accumulation error did not depend on it. The reason is that RPLD position for point dose measurement was placed in a plateau dose distribution. The dose distribution and RPLD position impacted on the DIR‐based dose accumulation error. Previous studies^18^, ^19^, ^42^ showed that DIR‐based dose accumulation greatly depended on DIR accuracy. Even if DIR error was small, a large DIR‐based dose accumulation error may be observed due to the sharp dose distribution.

In terms of CBCT, although CBCT images were used for moving images in various clinical situations (e.g., IGRT and ART), CBCT images were of inferior quality compared to planning CT images due to noise, artifacts and poor contrast resolution. CBCT images did not provide correct Hounsfield units (HU) and could not be directly used for the dose calculation. There have been a number of studies investigating the accuracy of CBCT‐based dose calculation.^43^, ^44^, ^45^ Our study aims to evaluate the dosimetric impact to DIR error. If CBCT images were used, we could not clarify the dosimetric impact between the CBCT‐based dose calculation error or DIR error.

The correlations of DSC, MDA, and TRE with the DIR‐based dose accumulation error over all data were −0.07, 0.05, and −0.30, respectively, for tumor‐proxy and 0.001, 0.09, and 0.47, respectively, for rectum‐proxy. The correlation coefficient indicates a positive value because one variable increases in value, the other increases, while the correlation coefficient shows a negative value because one variable increases in value, the other decreases. These results showed that TRE had a more correlation with the DIR‐based dose accumulation error than DSC and MDA. This is because TRE was evaluated with a point‐based metric, whereas DSC and MDA were calculated with contour‐based metrics.^20^ Dose mapping accuracy was measured using a point‐to‐point mapping metric based on the deformation vector field.^46^ Our data showed no correlation between the DIR‐based dose accumulation error with DSC and MDA. Shi et al.^46^ indicated that most contours‐based metrics had no correlation with DVF errors. Hence, well‐performed contour propagation does not directly indicate accurate dose deformation and summation within each contour. Their result was consistent with our result.

Our phantom was simple and it was not an anthropomorphic phantom. However, this phantom was designed to simulate anatomical changes using multiple custom inserts (e.g., tumor shrinkage and rectal filling), it could reproduce various deformation patterns. Moreover, our phantom was fabricated to be easy to change the deformation patterns. Several studies have shown that the anthropomorphic phantoms were created to evaluate DIR tools.^47^, ^48^ The phantom of Kirby et al consisted the deformable tissue, bony anatomy and void (pharynx) for a head and neck region. Inflation and deflation of a balloon catheter inside the phantom simulated tumor shrinking. The head and neck phantom of Singhrao et al was constructed from rigid (bone) and deformable (soft tissue) materials that mimicked HU values for actual anatomy. The head to flex backward simulated a slightly different head position. Although the both anthropomorphic phantoms were created based on real patient anatomy, they might not generate several deformation patterns.

The AAPM TG 132 had recommended a DSC value of 0.8−0.9, an MDA value of 2−3 mm and a TRE value of 2−3 mm as the acceptance criteria.^20^ However, the acceptance criteria for dose accumulation had not yet been defined. The use of deformable registration for dose accumulation is outside of the scope of this task group. It is recommended that these issues be addressed in a subsequent task group. Protocols should be defined to guide the process for each treatment site, accounting for expected uncertainties and ensure detection of unexpected levels of uncertainties. The proposed phantom had simple structures but the clinical situation used patient anatomy had complex structures. Thus, the DIR accuracy of the proposed phantom should be higher than that of the clinical situation. However, this phantom combined with easy and difficult deformation patterns a likely in clinical practices. Consequently, the DIR accuracy of this phantom showed that some deformation pattern was high but some deformation pattern was low.

The present study has some limitations. First, we used only three DIR software: Velocity, RayStation and elastix. It would be preferable to use many types of DIR software. Second, we evaluated only one clinical site: the pelvis. Third, we created only two patterns because there were only two tumor‐proxy custom inserts and four rectum‐proxy custom inserts into which RPLDs could be inserted for dose measurement. Fourth, we did not account the RPLD density in dose calculation because the RPLD is very small volume to compare with whole phantom. Thus, the RPLD density had a small effect on the dose error. Fifth, the entire phantom was scaled down uniformly for the phantom shrinking test. The purpose of a scaled‐down phantom was to simulate patient weight loss but this situation may represent an unlikely in actual clinical practices (the bony anatomy was not shrunk but the surrounding soft and adipose tissues were shrunk by weight loss). This test was designed to increase severe condition for deformation of the phantom. In the future, we plan to develop the phantom a likely in clinical scenario. As an example, the phantom was created from deformable tissue and bony anatomy.

## 5 CONCLUSIONS

We developed new custom inserts in a physical geometric phantom into which RPLDs were inserted for dose measurement. The DIR‐based dose accumulation error was 3% for tumor‐proxy and less than 5% for rectum‐proxy, which was clinically acceptable. The custom‐developed physical phantom with inserts showed potential for accurate evaluation of DIR‐based dose accumulation. The prospect of simultaneous evaluation of geometric and dosimetric DIR accuracy in a single phantom may be useful for validation of DIR for clinical use. Similar methods or phantom design or RPLD based dosimetry can be used to evaluate the DIR based dose accumulation accuracy.

## AUTHOR CONTRIBUTION

Siwaporn Sakulsingharoj: performing the experiments, analyze data, and writing original draft and editing. Noriyuki Kadoya and Mitsuhiro Nakamura: conceptualization, funding acquisition, and writing review and editing. Shohei Tanaka and Kiyokazu Sato: helping the experiments, and Keiichi Jingu: provision of study resources.

## CONFLICT OF INTEREST

We have no conflicts of interest to disclose.
